# Chemokine Profile and the Alterations in CCR5-CCL5 Axis in Geographic Atrophy Secondary to Age-Related Macular Degeneration

**DOI:** 10.1167/iovs.61.4.28

**Published:** 2020-04-23

**Authors:** Marie Krogh Nielsen, Yousif Subhi, Christopher Rue Molbech, Mads Krüger Falk, Mogens Holst Nissen, Torben Lykke Sørensen

**Affiliations:** 1 Clinical Eye Research Division, Department of Ophthalmology, Zealand University Hospital, Roskilde, Denmark; 2 Department of Clinical Medicine, University of Copenhagen, Copenhagen, Denmark; 3 Eye Research Unit, Department of Immunology and Microbiology, University of Copenhagen, Copenhagen, Denmark

**Keywords:** age-related macular degeneration, geographic atrophy, neovascular AMD, chemokine, chemokine receptor

## Abstract

**Purpose:**

Geographic atrophy (GA) secondary to age-related macular degeneration (AMD) is a progressive disease with no treatment option. Previous studies show chemokine-mediated recruitment of immune cells in the retina, and therefore we investigated systemic levels of chemokines and chemokine receptors in patients with GA.

**Methods:**

This observational prospective study was conducted at a single center. We included 122 participants with no immune disease: 41 participants with GA and no choroidal neovascularization, 51 patients with neovascular AMD, and 30 healthy control individuals. Flow cytometric analysis was used to detect expression level of C-C chemokine receptor (CCR)1, CCR2, CCR3, CCR5, and C-X-C motif chemokine receptor (CXCR)3 on peripheral blood mononuclear cells (CD14+ monocytes, CD4+ T cells, CD8+ T cells). Plasma levels of C-C motif ligand (CCL)11, C-X-C motif chemokine (CXCL)10, and CCL5 were measured by specific immunoassays. Enlargement rate of GA lesion was measured from autofluorescence images.

**Results:**

Participants with GA have a specific chemokine profile with a higher expression of CCR5 than healthy controls in peripheral blood mononuclear cells, and a higher plasma levels of CCL-5. Further, GA was associated with higher monocytic expression of CCR2 than in neovascular AMD. We found that a high expression level of CCR5 on CD8+ T cells was associated with slower enlargement rate of atrophic lesion.

**Conclusions:**

The study showed an association between systemic chemokine profile and GA formation. Further studies are needed to fully elucidate the possible role of systemic chemokine regulation in mediating pathogenesis of GA.

Age-related macular degeneration (AMD) is a prevalent multifactorial disease, characterized by degeneration of the retinal tissue and progressive loss of visual function. Two late stages are described, which sometimes are seen in conjunction. The neovascular late stage with formation of choroidal neovascularization (CNV), leading to leaking and bleeding into the retinal layers, and resulting in the formation of fibrovascular scar tissue. The atrophic late stage, termed geographic atrophy (GA), is seen as extending atrophic lesions involving the outer photoreceptor layer, retinal pigment epithelium (RPE), and choriocapillaris. These atrophic lesions often form parafoveally and expand gradually to ultimately involve the fovea.^1^ Morphologically, the two late stages of AMD are very distinct, and in this study, we chose to include patients with GA only or CNV to compare these subgroups from an etiological perspective.

The involvement of immune cells in the pathology of AMD is widely accepted. Infiltrating immune cells have been shown in donor retinas and choroids of AMD patients,^2^ and several studies have shown the pivotal involvement of chemokines in this recruitment.^3^^,^^4^ Immune cell infiltration holds a homeostatic role in the resolution of inflammation. During inflammation, recruitment of phagocytic immune cells can clear toxic extracellular material, and thereby relieve tissue stress and preserve tissue function.^5^ However, late stage AMD is characterized by the chronic presence of subretinal immune cells in large drusen, in the advancing junctional zone of atrophic lesions, and in CNVs. Hence these cells are highly suspective to play a central role in disease progression.^6^ Therefore we included GA progression rate as a secondary outcome to explore any association between chemokine expression on different cell types and the individual loss of retinal tissue.

Cells of the innate immune system (monocytes, macrophages, dendritic cells, and natural killer cells) and the adaptive immune system (CD4+ T cells, CD8+ T cells, and B cells) are characterized by the constant mobility from the blood and lymphatic system into and within tissue. Chemokines and their receptors have key roles in the movement of immune cells and are implicated in a wide range of inflammatory diseases involving the central nervous system.^7^ One chemokine can bind to several receptors, and receptors are sensitive to several ligands.^8^ C-C chemokine receptor (CCR)1 and CCR2 have been found to be upregulated in CD14+ CD16+ monocytes in patients with neovascular AMD.^9^ CCR2+ monocyte cells have been shown to infiltrate the subretinal space in the atrophic lesions in donor eyes with GA.^4^ Further, C-C motif ligand (CCL)2-CCR2 interaction has been implicated in CNV formation.^10^ The C-X-C motif chemokine receptor (CXCR)3 has important inhibitory functions in angiogenesis and has been found to be expressed at lower levels on CD8+ T cells in patients with neovascular AMD.^11^ Further, its ligand, C-X-C motif chemokine (CXCL)10, is found increased in aqueous humor from patients with AMD.^12^ CCR3 is expressed in choroidal neovascular cells, in which signaling with ligand CCL11 leads to proliferation and CNV growth. Blocking of this signaling has been suggested as a treatment target in neovascular AMD.^13^ CCL5, also known as RANTES (regulated on activation, normal T cell expressed and secreted), is a chemotactic cytokine that regulates inflammatory cell migration, is suggested to influence the course of AMD through secretion by RPE cells.^14^ CCL5 is implicated in other age-related neurodegenerative diseases, such as Parkinson disease.^15^

In this study, we aimed to map alterations in chemokine levels and chemokine receptor expression on circulating immune cells, CD14+ monocytes, CD4+ T cells, and CD8+ T cells from patients with GA, and to explore whether chemokine receptor levels were associated with GA enlargement rate.

## Methods

### Participants and Inclusion

This was a prospective case–control study. The study adhered to the tenets stated in the Declaration of Helsinki and was approved by the Regional Committee on Health Research Ethics (journal no. SJ-385).

All patients were recruited from the retinal outpatient program at Zealand University Hospital, Roskilde, Denmark. Patient spouses participated as healthy controls to best match patients in terms of age and lifestyle choices. All participants had the nature of the study explained verbally and written, and gave informed consent prior to participation.

Participants were subjected to a structured interview regarding past medical history, medication use, tobacco and alcohol use, and a single sentence question was used to assess regular physical activity.^16^ Participants were not included if they had any ongoing infectious or inflammatory disorders, used any immune modulating medication, or suffered from any cancer disease. Participants with C-reactive protein (CRP) level above 15 mg/L were excluded post hoc, due to suspected acute inflammation, as studies have found that in healthy aged individuals, 15 mg/L represents the 99th percentile.^17^

Fresh blood sample was obtained at initial visit into one vacutainer coated with ethylenediaminetetraacetic acid for flow cytometry, and two vacutainers coated with lithium-heparin for plasma isolation and for measurement of CRP.

### Retinal Diagnosis

All participants, both patients with AMD and healthy controls, underwent detailed ophthalmological examination of both eyes, including best corrected visual acuity according to the protocol Early Treatment Diabetic Retinopathy Study,^18^ indirect dilated fundoscopy, digital fundus photography, spectral domain optical coherence tomography, and fundus autofluorescence image. All examinations were performed by a retinal specialist. Retinal angiography using fluorescein and indocyanine green was used only in cases of suspected CNV to secure diagnosis of neovascular AMD.

Eyes were graded according to the Clinical Age-Related Maculopathy Grading System (CARMS),^19^ and patients were included in the following categories:

• Healthy controls had no ocular disease, less than 10 small drusen (<63 µm), no pigment abnormalities, equivalent to CARMS grade 1, in both eyes.
• Patients with GA had sharply demarcated areas of atrophy in the macular area, involving the RPE and outer photoreceptor layer in one or both eyes. Equivalent to CARMS grade 4. Patients in this group did not have any history or clinical signs of any former CNV.
• Patients with neovascular AMD had nondrusenoid pigment epithelial detachments, serous retinal detachments, and choroidal neovascular membrane with subretinal fluid, hemorrhages or fibrosis in one or both eyes, equivalent to CARMS grade 5.

### Chemokine Receptor Expression

We performed flow cytometric analysis on venous blood samples within 4 hours of phlebotomy. White cell count was determined using the automated hematology analyzer system Sysmex KX-21N (Sysmex Corporation, Kobe, Japan) to calculate the blood volume equivalent to a constant final number of leukocytes in the test tube (5 ×·10^5^ leukocytes). Red blood cell lysis buffer (Nordic Biosite AB, Täby, Sweden) was added, and the blood volume was lysed for 10 minutes at room temperature in the dark. The cells were washed three times using isotonic buffer solution (BD Biosciences, Franklin Lakes, NJ, USA), the suspension was centrifuged for 5 minutes at 500*g*, decanted and resuspended. Samples were aliquoted into two identical tubes. Fluorochrome-coupled antibodies were added to one tube, and corresponding isotype controls of each antibody was added to the second tube (Supplementary Table S1). Both tubes were incubated in the dark at room temperature, washed and resuspended in isotonic buffer. Cells were analyzed within 4 hours of phlebotomy using the BD FACSCanto II flow cytometer (BD Biosciences) with a sample size of 100,000 singlet leukocytes using a preset gate. Final analysis was performed using Kaluza Software v. 1.5.20365.16139 (Beckman Coulter, Inc., Pasadena, CA, USA).

All cells were plotted on a forward scatter area versus forward scatter height to select singlet cells. Monocytes and lymphocytes were identified from their size and complexity shown in a forward scatter area versus side scatter area. Lymphocytes were divided into subsets according to their expression of CD4 and CD8. Monocytes were gated by CD14 expression. Individual chemokine receptor expression was determined for each cell type from fluorescence intensity of each fluorochrome-specific antibody, with negative isotype controls set at 1% threshold. Gating strategy is shown in Supplementary Figure S2. Two investigators performed flow cytometric analysis blinded from diagnosis or any clinical or demographic information.

### Plasma Chemokine Concentration

One lithium-heparin coated tube was centrifuged for 15 minutes at 1500*g* to eliminate cells. Plasma was immediately pipetted, aliquoted, and stored at –80°C. Plasma levels of CCL11, CXCL10, and CCL5 were measured using commercially available multiplex immunoassays (Meso Scale Discovery, Gaithersburg, MD, USA). Samples were diluted 50-fold, plates were prepared according to manufacturer's instructions, and reading was performed immediately on QuickPlex SQ120 (Meso Scale Discovery). Concentrations were calculated from the eight-point standard curve in each plate. All samples were run in duplicate and performed randomly across plates, and investigators (MKN and YS) were blinded from diagnosis. The coefficient of variance (CV) was calculated between each duplicate test, and tests were repeated if the CV value exceeded 20%. The quality of each chemokine assays was satisfactory (CV in % of mean ± SD: CCL11: 3.2 ± 2.4; CXCL10: 3.3 ± 2.3; CCL5: 3.7 ± 3.3).

### Enlargement Rate of Atrophic Lesion in GA

Patients with GA were invited for follow-up examination after 1 year from inclusion. The procedures used for analysis and grading of autofluorescence images have been published elsewhere.^20^

### Data Analyses

All analyses were performed using the Statistical Package for the Social Sciences (IBM Corporation, Armonk, NY, USA). Normally distributed data are presented using mean and standard deviation (SD), and compared using parametrical test. Not normally distributed data are presented using median and interquartile range and compared using nonparametrical tests. Categorical data are compared using the χ^2^ test. Associations were described using univariate linear regression. *P* values below 0.05 were interpreted as statistically significant, and effect size was estimated after calculation of Cohen's *d*, interpreted as suggested by Cohen: >0.2 small, >0.5 moderate, >0.8 large.^21^

## Results

### Participants

A total of 127 participants were enrolled in the study. Five individuals were excluded post hoc due to elevated levels of CRP: one healthy control (CRP = 17 mg/L), one participant with GA (CRP = 20 mg/L), and three participants with neovascular AMD (CRP = 17, 18, and 70 mg/L). Therefore 122 participants were included in the final analysis: 41 participants with GA, 30 healthy control individuals, and 51 participants with neovascular AMD.

Participants did not differ significantly in demographic background, lifestyle, or comorbidities, as presented inTable 1^2^. Patients with any AMD had a tendency toward higher occurrence of hypertension (*P* = 0.092), and cardiovascular disease (*P* = 0.076). This is in line with current literature, describing hypertension and cardiovascular disease as associated with late-stage AMD.^22^ Similarly, there was a tendency toward higher levels of CRP in patients with AMD (*P* = 0.055), in particularly in patients with GA. These changes demonstrate that the selected population is representative as CRP, as well as other markers of chronic inflammation is known to be elevated in late-stage AMD.^20^^,^^23^ The patients with neovascular AMD had the disease for a median period of 20.5 months (range, 1–92). All patients with neovascular AMD received treatment with regularly administered intravitreal anti-vascular endothelial growth factor. Criteria for retreatment was based on the Danish Ophthalmological Society guideline and recommendations.^24^

**Table 1.** Participant Characteristics

	Diagnosis			
	GA, *n* = 41	Healthy Controls, *n* = 30	Neovascular AMD, *n* = 51	*P* Value
Age, y (SD)	78.4 (6.5)	75.6 (6.1)	75.8 (7.4)	0.129*
Sex, no. (%)				
Female	26 (63)	15 (50)	26 (51)	0.430^†^
Male	15 (37)	15 (50)	25 (49)	
Smoking, no. (%)	11 (27)	3 (10)	15 (29)	0.353^†^
Current				
Previously	17 (42)	15 (50)	22 (43)	
Never	13 (32)	12 (40)	14 (28)	
Alcohol, median (IQR)	4.0 (2.0–8.0)	4.0 (2.0–7.0)	3.0 (0.5–8.5)	0.565^‡^
Body mass index, mean (SD)	26.7 (6.0)	25.7 (3.6)	26.1 (4.0)	0.706^*^
Exercise, no. (%)	26 (63)	18 (64)	27 (53)	0.480^†^
Hypertension, no. (%)	24 (59)	10 (33)	27 (53)	0.092^†^
Hypercholesterolemia, no. (%)	14 (34)	9 (30)	13 (26)	0.674^†^
Cardiovascular disease, no. (%)	15 (37)	4 (13)	12 (24)	0.076^†^
Type 2 diabetes, no. (%)	6 (15)	0	6 (12)	0.116^†^
CRP, mg/L				
<2.9, no (%)	23 (56)	26 (87)	32 (63)	0.055^§^
3–10, no (%)	15 (37)	4 (13)	14 (27.5)	
10–15, no (%)	3 (7)	0	5 (10)	

IQR, interquartile range.

* One-way ANOVA.

† χ^2^ test.

‡ Kruskal-Wallis test.

§ Fisher's Exact test.

**Table 2.** Chemokine Receptor Expression on Peripheral Blood Mononuclear Cells, Reported as Percentage of Positive Cells from the Given Population. Chemokine Concentrations are Measured in Plasma. Measures are Reported as Median and Interquartile Range.

	Diagnosis			*P* Value^*^		
	GA, *n* = 41	HC, *n* = 30	nAMD, *n* = 51	GA vs. HC	GA vs. nAMD	HC vs. nAMD
*CD14+ monocytes*						
CCR1	36.4 (29.8–51.8)	27.1 (1.8–55.8)	36.9 (19.0–62.5)	0.345	0.957	0.459
CCR2	93.7 (90.5–96.9)	92.2 (84.6–96.6)	91.9 (83.5–95.7)	0.068	**0.042**	0.969
CCR5	1.2 (0.9–1.9)	0.5 (0.2–1.3)	1.0 (0.5–1.6)	**<0.001**	**0.009**	0.180
CXCR3	0.85 (0.6–1.9)	0.5 (0.3–0.9)	0.8 (0.5–1.5)	**0.021**	0.370	**0.047**
*CD4+ T cells*						
CCR1	0.2 (0.1–7.7)	0.2 (0.1–0.7)	0.2 (0.1–0.3)	0.122	0.074	0.704
CCR2	6.1 (3.9–9.9)	5.5 (3.5–10.6)	5.8 (3.8–8.9)	0.618	0.749	0.799
CCR5	2.5 (1.3–4.6)	1.1 (0.7–3.6)	1.8 (1.0–3.6)	**0.024**	0.190	0.225
CXCR3	4.3 (1.1–11.4)	2.9 (0.8–6.9)	2.7 (1.8–4.5)	0.367	0.270	0.845
*CD8+ T cells*						
CCR1	0.6 (0.2–2.6)	0.3 (0.1–1.0)	0.2 (0.2–0.9)	0.213	0.294	0.795
CCR2	6.8 (4.9–11.3)	7.2 (5.8–11.8)	8.1 (5.7–11.3)	0.906	0.576	0.681
CCR5	11.5 (6.1–16.3)	5.1 (2.8–12.2)	10.3 (4.2–17.0)	**0.031**	0.603	0.087
CXCR3	5.7 (1.1–16.2)	5.0 (1.0–11.2)	4.2 (1.8–8.5)	0.506	0.502	0.992
*Plasma chemokine concentration*						
CCL11, pg/mL	951 (783–1161)	881 (741–1064)	988 (736–1239)	0.442	0.950	0.401
CXCL10, pg/mL	513 (393–732)	430 (330–560)	478 (308–585)	0.073	0.217	0.532
CCL5, pg/mL	42,115 (27,922–56,108)	29,528 (24,791–37,174)	27,568 (18,887–39,708)	**0.017**	**0.001**	0.484

HC, healthy control; nAMD, neovascular age-related macular degeneration.

Bold values indicate *P*-value ≤ 0.05.

* Mann-Whitney *U* test.

### Chemokine Receptor Expression

Patients with GA had an increased proportion of CCR5+ peripheral blood mononuclear cells compared with healthy controls. Monocytic expression of CCR5 was significantly increased in patients with GA, with a small effect size (*P* < 0.001, Cohen's *d* = 0.3) and in patients with neovascular AMD (*P* = 0.009, Cohen's *d* = 0.2) compared with healthy controls. Patients with GA also had a significantly higher expression of CCR5 in CD8+ T cells, (*P* = 0.024, Cohen's *d* = 0.4) and in CD4+ T cells, in which the difference was found to have a moderate effect size (*P* = 0.031, Cohen's *d* = 0.5). Expression of CCR5 on T cells did not differ between patients with neovascular AMD and healthy controls.

Patients with GA had increased expression of CCR2 on CD14+ monocytes compared with patients with neovascular AMD (*P* = 0.042, Cohen's *d* = 0.6), and to a lesser extent compared with healthy controls (*P* = 0.068, Cohen's *d* = 0.6).

Expression of CXCR3 was found to be increased on CD14+ monocytes both in patients with GA (*P* = 0.021, Cohen's *d* = 0.4) and in patients with neovascular AMD, although the effect size was small (*P* = 0.047, Cohen's *d* = 0.1). Expression is given in Table 2 and distribution shown in Supplementary Figure S3.

### Plasma Chemokine Levels

Patients with GA had significantly higher plasma levels of CCL5, both compared with healthy controls (*P* = 0.017) and patients with neovascular AMD (*P* = 0.001). Effect size of this difference showed a moderate size (Cohen's *d* = 0.7). Plasma levels of CCL11 and CXCL10 did not differ across groups, however, there was a tendency toward higher CXCL10 among patients with GA (*P* = 0.073).

### Association with GA Progression During One Year

Considering that chemokine-mediated recruitment of peripheral immune cells are known to play a role in late stage AMD, but their exact role remains unknown, we chose to test if a possible association existed between the altered levels of chemokine expression levels and the progression rate of GA. All patients with GA were invited for a follow-up visit 1 year later, and from the 41 participants, 32 patients completed follow-up examination on average after 13 months from baseline (range, 12–18 months). Overall GA enlargement rate was median 1.64 mm^2^/year (interquartile range, 0.95–2.17). After correction for baseline area (square root transformation), we plotted each of the measured receptors, as shown in the Figure. We found that the proportion of CCR5+ CD8+ T cells associated with enlargement of GA lesion (R^2^ = 0.154, *P* = 0.018) in a univariate model, with high expression of CCR5 being indicative of slower progression (Slope = –0.22; 95% confidence interval, −0.40 to −0.04).

**Figure.**
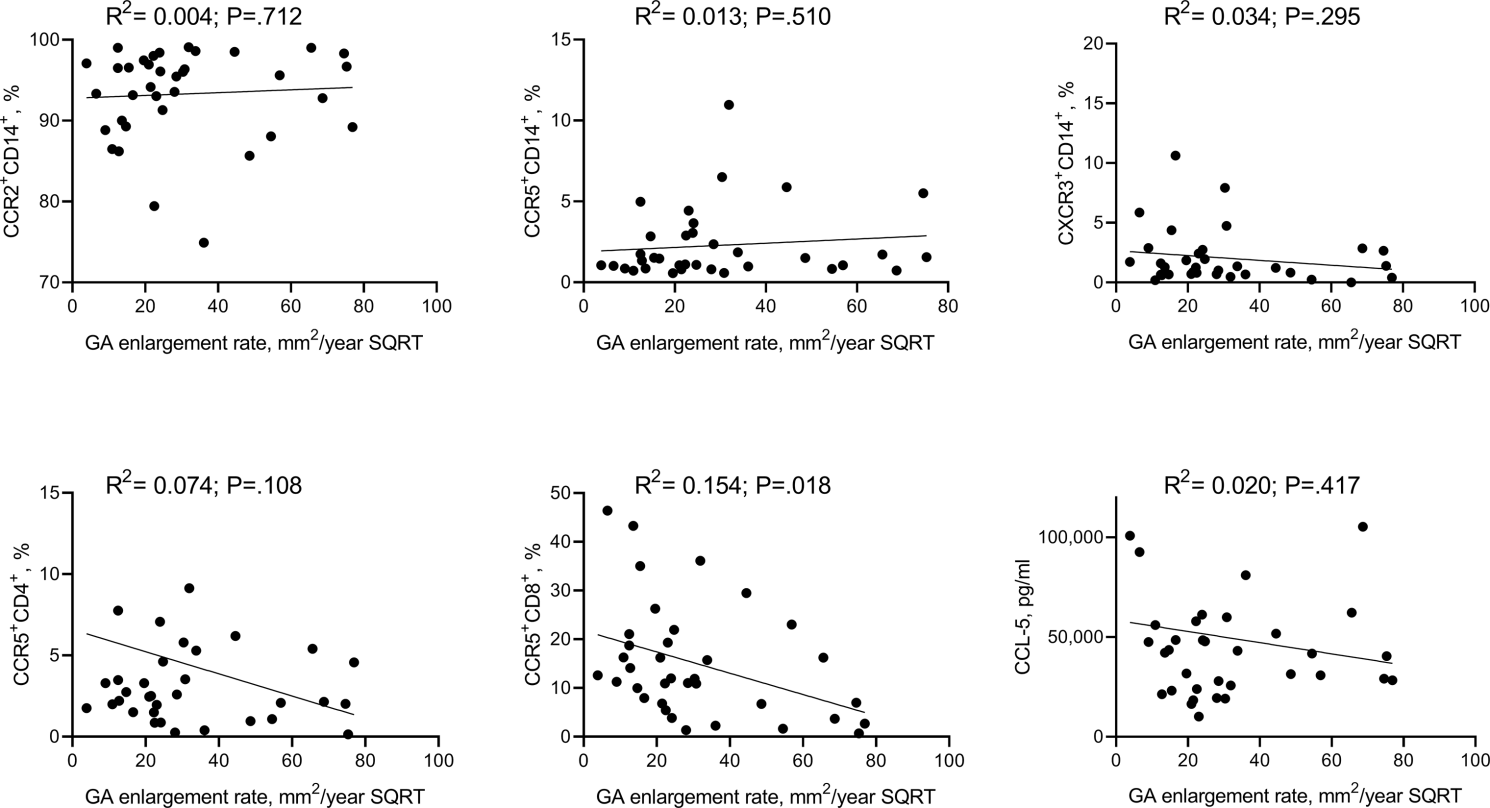
Enlargement rate of GA during 1 year was square root transformed (SQRT) for baseline area and plotted against plasma levels of CCL5, CCR5+ CD14+ monocytes, CCR2+ CD14+ monocytes, CXCR3+ CD14+ monocytes, CCR5+ CD4+ T cells, and CD8 CCR5+ T cells). This revealed a moderate but significant association between progression rate and CCR5 expression on CD8+ T cells.

## Discussion

This study presents a peripheral chemokine profile for patients with GA. Our findings strongly suggest a novel role for the chemokine/chemokine receptor axis CCL5/CCR5 in GA. We found that patients with GA had increased expression of CCR5 on peripheral blood mononuclear cells. The involvement of CCR5 in GA and AMD is not previously described, but CCR5 is known to play a central role in other age-related neurodegenerative diseases. Further, we find increased monocytic expression of CCR2. This is in line with current literature, describing CCR2-mediated recruitment of blood-derived mononuclear phagocytes as a central part of disease mechanism.^6^ CCL2 is increased in eyes with GA, and specifically found in the vessels and in the outer retina adjacent with the atrophic lesion. Further, CCR2+ monocytes are recruited and accumulated in the subretinal in patients with GA. In an animal model of AMD, CCL2 deficiency protects from photoreceptor degeneration.^4^ This add to the notion that the CCR2-mediated recruitment of mononuclear phagocytes are contributing to the progressive loss of retinal tissue in GA.

The proinflammatory chemokine CXCR3 has been shown to possess angiostatic and antifibrotic properties via the binding of its ligand CXCL10.^25^^,^^26^ Interestingly, we find increased expression of CXCR3 on monocytes in both patient groups, but the tendency toward higher CXCL10 (*P* = 0.073) was only found in patients with GA, the late-stage characterized by the complete absence of angiogenesis and fibrosis.

The chemokine receptor CCR-5 is widely expressed at the cell surface of antigen-presenting cells (APCs). Effector T cells and NK-cells produce CCR-5 ligands on binding of APCs to their targets. Production of these ligands attracts more CCR-5+ effector cells to the site. CCR-5 is particularly important in the biology of CD8+ T cells, as it controls their priming and maturation.^27^ Naive T cells do not express CCR-5, but this is upregulated on CD8+ T cells on their entry into lymph nodes. Effector T cells are guided to sites of inflammation by a gradient of chemokines. Microglia, astrocytes, and neurons are able to produce the CCR5 ligands, CCL3, CCL4, and CCL5, participating in a multistep adhesion cascade by triggering CCR5 and activating cell surface integrins, promoting transendothelial migration of circulating CCR5+ effector T cells. CCR5-CCL5 interaction has been shown to play a key role in several neuroinflammatory diseases, such as multiple sclerosis, Rasmussen encephalitis, and cerebral malaria, in which CD8+ CCR5+ T cells infiltrate the brain, leading to neuronal destruction.^28^^,^^29^ However, the physiological functions of CCR5 in the central nervous system are not fully understood, as CCR5-CCL5 interaction has also been shown to hold a neuroprotective role. Loss of dopaminergic neurons has been reported in CCR5 deficient mice,^30^ and CCL5 promotes neuronal survival in proapoptotic conditions,^31^ and protects against certain encephalitis virus.^32^^,^^33^

We find CCR5 expression on CD8+ T cells to be negatively correlated with GA enlargement rate, indicating that in this particular disease, CCR5-CCL5 interaction might hold a neuroprotective role. When considering that GA is a disease characterized by low-grade chronic inflammation,^20^ it seems likely that the role of CCR5-CCL5 interaction differs from the role seen in diseases, such as Rasmussen encephalitis and cerebral malaria, characterized by an acute inflammatory response.

We find an overall low expression of CCR5 on CD14+ monocytes, which is not surprising because CCR5 is not expressed on circulating monocytes under homeostatic conditions. However, CCR5 is expressed on monocytes during inflammation, and CCR5+ monocytes play a crucial role in orchestrating an appropriate sepsis immune response.^34^ In this study, CCR5 expression on monocytes is slightly increased in patients with late-stage AMD, which is in line with the association between both late stages of AMD and systemic low-grade chronic inflammation, with the highest levels in patients with GA.^20^^,^^23^

Plasma levels of CCL5 increases as a function of age and “inflammaging,”^35^ therefore it is not surprising to find this chemokine increased in aged individuals and in patients with GA, whom we have previously found to have accelerated immunological aging and chronic inflammation.^20^ Rentzos et al.^36^ reports a similar increase in plasma CCL5 in patients with Parkinson disease. Interestingly, one study developed an in vitro blood–brain-barrier using SV40 T-antigen immortalized human brain microvascular endothelial cells, which did not secrete CCL5 under homeostatic conditions, but when stimulated with proinflammatory cytokines there was a dose-dependent increase in CCL5 production by the cells, simultaneously with a marked degraded barrier function.^37^^,^^38^ Ageing and the associated chronic inflammation has also been shown to trigger CCL-5 and CCL-7 release by human retinal pigment epithelial cells and choroidal fibroblasts,^39^ but whether this release is protective or harmful is debated. In vitro, CCL5 was able to suppress expression of inflammatory cytokines (IL-1β, IL-6, and TNF-α) in microglia. This suppression was not seen in CCR5-knockout mice, suggesting that CCR5-signaling protects neurons by suppressing microglia toxicity.^40^ This is of particular interest, as inappropriate microglia activation is another key feature in GA pathogenesis.^41^^,^^42^

Several limitations should be kept in mind when interpreting these results. The observational nature of this study merely provides us with associations between events, such as CCR5 expression and GA progression. However, a study with a larger sample size is necessary to provide robust data on the course of disease.^43^ Also, experimental studies are needed to evaluate the causality of these events. The two groups of late AMDs—GA and neovascular AMD—obviously differ in terms of neovascularization, but they also differ in the treatment with anti-vascular endothelial growth factor. We are unaware if these antibodies might influence the systemic levels of chemokines or chemokine receptor expression in these patients. We did not include treatment naive patients with neovascular AMD, as the initial CNV-formation is associated with an acute immune response.^44^ Further, we performed a small single-center study, and larger studies are needed to perform multivariate analysis, or to investigate the influence of GA subtypes on chemokine profile.

## Conclusions

AMD is an increasingly prevalent disease, which will continue to affect visual health, daily functions, and quality of life among thousands of elderly individuals. We find that patients with GA have an altered chemokine profile, which might be associated with disease progression. Chemokine modulation might provide hope for a means against disease development and progression of GA, which currently holds no treatment option.

## Supplementary Material

Supplement 1

Supplement 2

Supplement 3
