# Supplementary material for: Chemokine Profile and the Alterations in CCR5-CCL5 Axis in Geographic Atrophy Secondary to Age-Related Macular Degeneration
Source: Invest Ophthalmol Vis Sci. 2020 Apr 23;61(4):28. doi: 10.1167/iovs.61.4.28 (PMC7401724; doi:10.1167/iovs.61.4.28)
Supplement: Supplement 1 [file iovs-61-4-28_s001.pdf]

Supplementary Table 1. Antibodies used for flow cytometry

**Target**	**CD14**	**CD4**	**CD8**	**CCR1**	**CCR2**	**CCR3**	**CCR5**	**CXCR3**
Fluorochrome	Pacific Blue	PerCP	Brilliant Violet	APC	PE	APC/Cy7	FITC	PE/Cy7
Isotype	IgG1	IgG2a	IgG1	IgG2b	IgG2b	IgG2b	IgG2b	IgG1
Clone	HCD14	#11830	RPA-T8	#43504	#48607	5E8	#45531	1C6
Manufacturer	BioLegend	R&D Systems	BioLegend	R&D Systems	R&D Systems	BioLegend	R&D Systems	BD Biosciences
**Negative isotype**								
Fluorochrome				APC	PE	APC/Cy7	FITC	PE/Cy7
Isotype				IgG2b	IgG2b	IgG2b	IgG2b	IgG1
Clone				MPC-11	#133303	MPC-11	#133303	MOPC-21
Manufacturer				BioLegend	R&D Systems	BioLegend	R&D Systems	BioLegend

Manufacturers: BD Biosciences, Franklin Lakes, NJ, USA; BioLegend, San Diego, CA, USA; R&D Systems, Minneapolis, MN, USA.

Abbreviations: APC = Allophycocyanin; PE = Phycoerythrin; Cy7: Cyanine-7; FITC: Flouresceinisothiocyanat.
