# Supplementary material for: Chemokine Profile and the Alterations in CCR5-CCL5 Axis in Geographic Atrophy Secondary to Age-Related Macular Degeneration
Source: Invest Ophthalmol Vis Sci. 2020 Apr 23;61(4):28. doi: 10.1167/iovs.61.4.28 (PMC7401724; doi:10.1167/iovs.61.4.28)

Figure 2. Gating strategy used to determine chemokine receptor expression in subtypes of peripheral blood mononuclear cells. A: From forward-scatter height (FSC-H) and forward-scatter area (FSC-A), we isolated singlet cells. B: Based on FSC-A and side-scatter area (SSC-A), representing cell size and complexity, we determined lymphocyte and monocyte populations. C: The lymphocyte population was differentiated in CD8+ and CD4+ cells, D: From the monocyte population, we isolated the cells that were CD14+. E: We quantified the proportion of each chemokine receptor expression on each cell type, stained with a specific monoclonal antibody (yellow) using a fluorescence histogram and a negative isotype control with a threshold of 1% (grey).

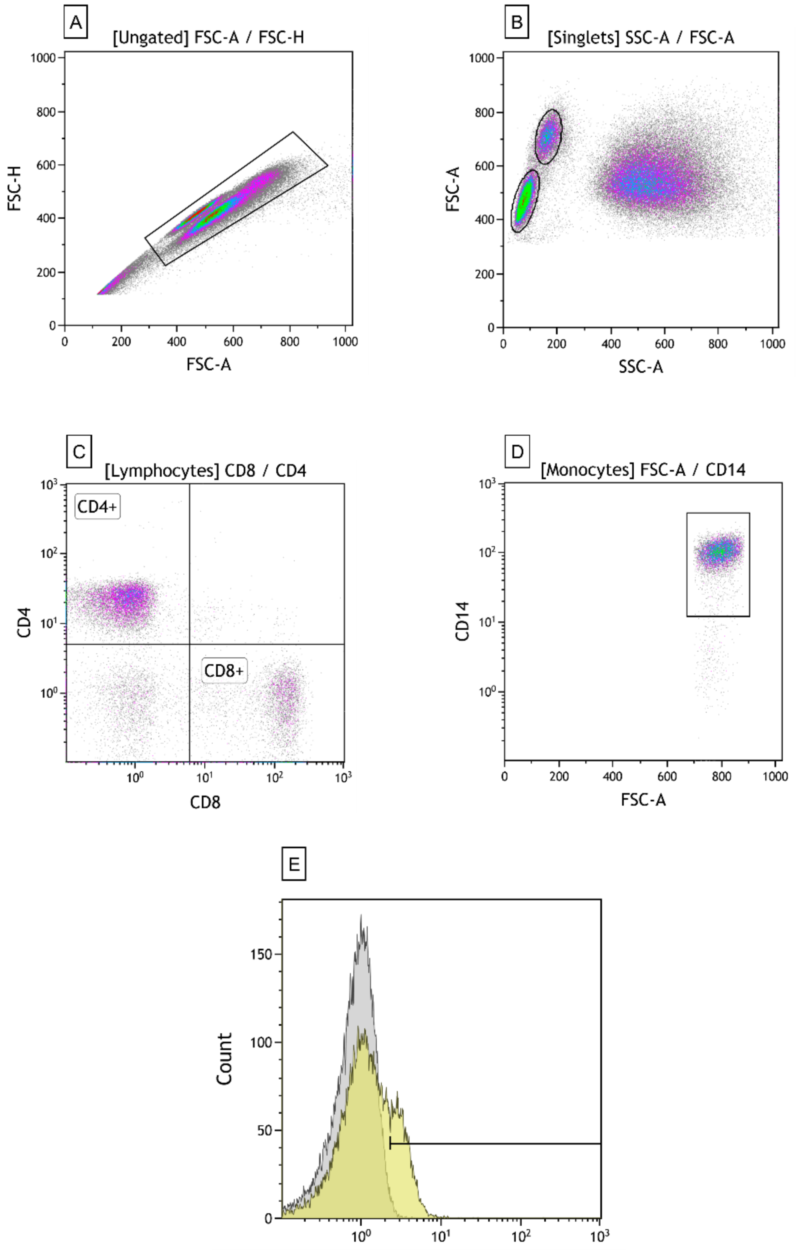

Supplement: Supplement 2 [file iovs-61-4-28_s002.pdf]
