# Supplementary material for: Chemokine Profile and the Alterations in CCR5-CCL5 Axis in Geographic Atrophy Secondary to Age-Related Macular Degeneration
Source: Invest Ophthalmol Vis Sci. 2020 Apr 23;61(4):28. doi: 10.1167/iovs.61.4.28 (PMC7401724; doi:10.1167/iovs.61.4.28)

Supplementary file 3. Distribution of significantly altered chemokine expression between healthy controls, patients with geographic atrophy (GA), and patients with neovascular age-related macular degeneration (nAMD).

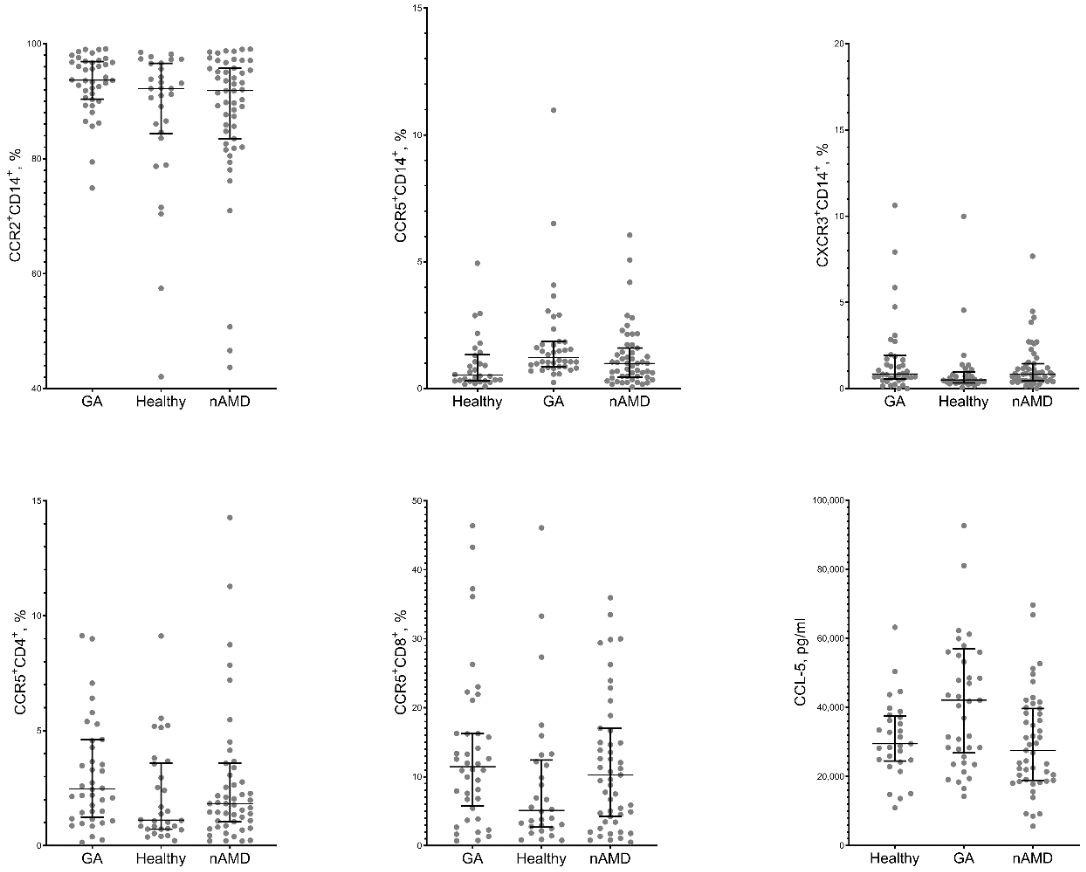

Supplement: Supplement 3 [file iovs-61-4-28_s003.pdf]
